# Exploring Composition and Within-Population Variation in the Phloem Exudate “Manna” in *Eucalyptus viminalis*

**DOI:** 10.3390/plants14152294

**Published:** 2025-07-25

**Authors:** Erin C. P. M. Bok, Geoffrey M. While, Peter A. Harrison, Julianne M. O’Reilly-Wapstra

**Affiliations:** School of Natural Sciences, University of Tasmania, Private Bag 55, Hobart, TAS 7005, Australia; geoffrey.while@utas.edu.au (G.M.W.); peter.harrison@ecoadapt.com.au (P.A.H.); julianne.oreilly@utas.edu.au (J.M.O.-W.)

**Keywords:** *Eucalyptus viminalis*, phloem exudate, carbohydrate allocation, seasonal variation, raffinose, sucrose, trait variation

## Abstract

Sugary phloem exudates are produced by many plant species and play key roles in carbon storage, defense, and ecological interactions. Among eucalypts, one such exudate, manna, is an important carbohydrate source for birds, mammals, and insects. Despite its ecological relevance, little is known about the composition and intra-specific variability of manna. Here, we investigated patterns of manna production in *Eucalyptus viminalis*, a widespread foundation tree species in southeastern Australia. We developed a repeatable ex situ method to extract and analyze manna, allowing us to characterize its sugar composition and examine variation within and between trees. Across years, manna contained six sugars, with sucrose and raffinose dominant. We found substantial variation in both the quality (sucrose/raffinose ratio) and quantity (mg) of manna produced. Both declined with increasing tree size (DBH), while quality increased with branch circumference. Seasonal and annual variation in manna was also evident, with quality increasing under drier conditions (positive correlation with aridity). Our findings demonstrate substantial intra-specific variation in phloem exudates (manna), shaped by temporal and tree-level factors. These patterns offer a foundation for future research into the ecological and physiological drivers of exudate variation and resource availability in foundation species like *E. viminalis*.

## 1. Introduction

Phloem exudates, such as manna, represent a carbon-rich output of plant metabolic processes and may reflect underlying physiological processes related to storage, defense, and environmental stress responses [1,2,3,4]. Despite their ecological relevance as a food source for many insects, birds and mammals, [5,6,7,8,9,10], few recent studies have investigated the composition and intra-specific variability of these exudates in wild plant populations [4,11,12].

Mannitol or mannite is a sap-like substance that occurs naturally in the phloem of most land plants as well as being produced by some bacteria, fungi, and yeast [1,13,14,15]. Mannitol production has been recorded in over 70 higher plant families including Oleaceae, Rubiaceae, Scrophulariaceae, and Apiaceae [2,15,16,17,18]. In plants, mannitol is extruded in response to small incisions by insects and birds in the leaves and young stems of the plant, which crystallizes into small white nodules referred to as “*manna*” [7,8,19,20]. Manna is a fructose-derived 6-carbon sugar alcohol (C_6_H_14_O_6_), composed predominantly of six sugars (raffinose, stachyose, sucrose, glucose, fructose, and tetrasaccharides) in addition to trace amounts of water, pectin, and uronic acids [13,19]. The composition and concentration of the six sugars has been found to vary within and between plant species [13,19]. Manna is most commonly associated with *Fraxinus* (Oleaceae) and *Eucalyptus* (Myrtaceae), where it is classified as a “dried exudate” [15,16,17,20]. Sucrose and raffinose, two of the dominant sugars in manna, have been proposed as key mediators of abiotic stress and carbon storage in plants, functioning as transportable reserves or protective solutes under environmental stress [1,2,3,4,19,21].

Within Australia, the *Eucalyptus* genus encompasses more than 800 species that occupy and dominate terrestrial habitat types, from dry deserts to tropical and temperate rainforests, and occur from sea level to the alpine tree line [22,23,24,25]. This broad occupation of various niches allows eucalypts to serve as foundation species, supporting biodiverse communities through the provision of food and habitat. Manna produced by *Eucalyptus* has been identified as a crucial food source for many native birds, mammals, and insects, offering a concentrated carbohydrate source [5,8,20]. Within the *Eucalyptus* genus, evidence of manna production exists in 14 species [19]. However, existing research has primarily focused on the feeding behaviors of insects and animals that rely on manna as a food source, with less emphasis on the variation in its composition [7,8,19,20]. It is established that the two dominant sugars sucrose and raffinose represent an alternate axis of quality for foragers, with sucrose being highly digestible and energy rich, whereas raffinose is indigestible [7,8,20,26]. However, knowledge gaps remain concerning how manna sugar composition, specifically in the context of these dominant sugars, varies within species and the abiotic and morphological drivers of this variation [4,12,27]. Investigating this variation in *Eucalyptus* species may shed light on underlying plant allocation strategies and help clarify how changes in carbohydrate composition influence ecosystem-level resource provisioning in eucalypt-dominated environments.

This study investigates patterns of variation in manna composition, quality, and quantity in the foundation tree species *Eucalyptus viminalis*, a key component of dry sclerophyll forests across Tasmania [24]. As a widespread and ecologically significant species that supports a diversity of fauna [26,28,29], *E. viminalis* is an ideal model for examining variation in manna. We hypothesized that manna composition, quality, and quantity would vary both temporally (seasonally and annually) and spatially (within and between trees), reflecting underlying physiological processes. At finer scales, local factors such as leaf area and branch position may also influence photosynthetic activity and carbohydrate allocation [30,31,32,33,34]. Between trees, differences in size and canopy health are likely to affect whole-plant sugar production and storage strategies [2,35,36,37,38]. Temporal variation in manna is also expected, given that sugar synthesis and partitioning in plants respond to seasonal changes in temperature, water availability, and photoperiod [1,2,4,12].

To investigate these hypotheses, we developed a standardized, repeatable protocol for direct manna extraction. While chemical methods exist [16,17,39], we adopted a simpler technique based on simulating natural incisions made by fauna [4,7,20]. Rather than applying this method in situ [40,41], we propose an ex situ approach using pruned branches in a controlled lab environment. This allows us to minimize variation due to external factors like insect herbivory, temperature, and humidity while maintaining consistent branch selection criteria (e.g., canopy position, aspect, and diameter). Although this design does not capture whole-plant transport dynamics, it provides a valuable baseline for assessing compositional differences in a controlled context.

Once the extraction protocol was established, we applied it to investigate tree-level and temporal drivers of manna variation. We defined composition as the relative profile of six key sugars (sucrose, raffinose, fructose, glucose, stachyose, and tetrasaccharides), quality as the ratio of the two dominant sugars, sucrose and raffinose, and quantity as the total mass of exudate produced per branch (mg). Using this approach, we aimed to (i) quantify within-population and within-tree variation in manna traits and assess how this variation is shaped by branch-level attributes; (ii) evaluate how manna composition, quality, and quantity relate to tree canopy health; and (iii) determine how these traits change across seasons and years. Our results will provide valuable information about resource provisioning within a foundation tree species, *E. viminalis*, and its dependent communities [8,20,26,28,29,42,43]. More broadly, it will contribute to a better understanding of how plant-level traits and temporal gradients drive functional variation in phloem exudate composition.

## 2. Results

### 2.1. Manna Composition

*Eucalyptus viminalis* manna was comprised of six key sugars that varied in both their abundance and their compositional contribution (Table 1). Specifically, manna was mostly comprised of two key sugars, sucrose and raffinose, which were detected in all samples and made up approximately 25% and 70% of the overall sample composition, respectively (Table 1). The remaining four sugars (glucose, fructose, stachyose, and tetrasaccharides) contributed approximately 3.12% collectively.

**Figure 1:**
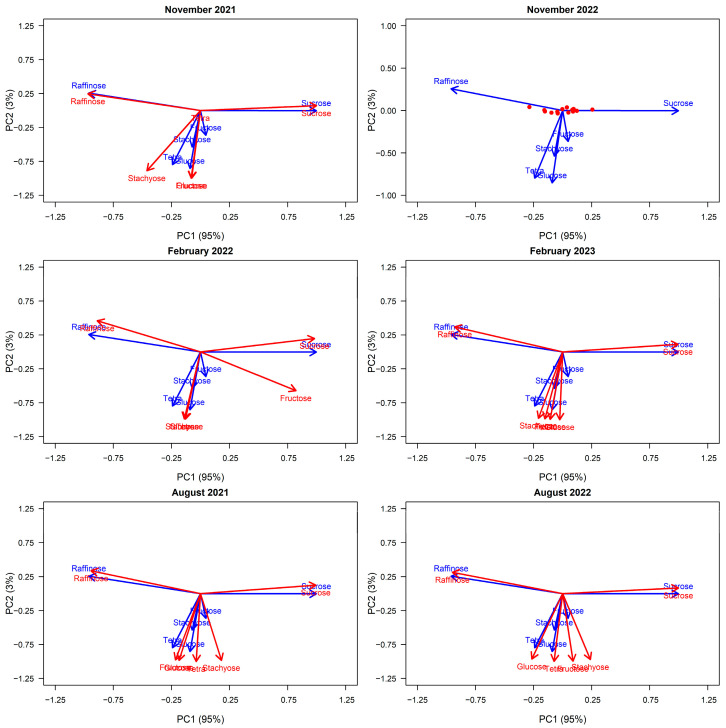
Principal component axes (PC1 and PC2) of the six key sugars in *E. viminalis* manna. PC1 and PC2 explain 95% and 3% of the variance, respectively. Arrows show sugar loadings; red dots indicate tree-level scores for the reference period (Nov 2022). The reference period is depicted in the top right panel; all other sampling periods (red) have been predicted into the November 2022 multivariate space (blue). Seasonally, August corresponds to winter, November to spring, and February to summer.

As shown in Figure 1, principal component analysis (PCA) of the six key sugars in *E. viminalis* manna collected in November 2022 revealed two main components that explained 98% of the variation in manna sugar composition. The first principal component (PC1) explained the majority of the variation in manna, accounting for 95% of the variance, and showed strong positive loadings for sucrose (1.00) and strong negative loadings for raffinose (−1.00) and tetrasaccharides (−0.72). PC2 accounted for 3% of the variance explained by the PC axes and represented three of the minor sugars, glucose (−0.78), stachyose (−0.56), and fructose (−0.29). PC3 represented < 2% of the variation in manna composition and, thus, was not considered further.

When projecting all other sampling periods into the November 2022 multivariate space, we observed consistent clustering of sugars across timepoints (Figure 1). Sucrose and raffinose consistently loaded along the primary axis (PC1), representing the dominant source of variation in manna composition, while the remaining minor sugars (fructose, glucose, stachyose, and tetrasaccharides) clustered more variably along PC2. This pattern suggests that sucrose and raffinose are the key drivers of compositional variation, and their inverse relationship is a stable feature across seasons. Seasonal differences in manna composition were instead most evident in the strength and orientation of the minor sugars along PC2, indicating subtle shifts in their relative abundances. Temporal changes in sugar configuration were assessed using a Procrustes test (Appendix E.1) to compare each sampling period to the November 2022 reference ordination. The test indicated dissimilarity in the arrangement of the six sugars across most timepoints (sum of squares = 0.747–0.964), suggesting changes in the relative positioning of sugars in the multivariate space. The exception was August 2022, which showed significant similarity to the reference period (*p* = 0.036). These results are consistent with visual observations that sucrose and raffinose dominate PC1 across all timepoints, while shifts in the configuration are largely driven by the minor sugars along PC2.

Given that sucrose and raffinose are both strongly represented on PC1 and together comprise ~90% of manna composition, subsequent analyses focused on these two sugars. Manna quality was represented by the sucrose/raffinose ratio, representing an alternate axis of nutritional value for foragers, and total manna weight (mg) as a measure of quantity. Although tetrasaccharides were represented in PC1, these sugars were excluded from the sucrose/raffinose quality metric due to its low abundance (<2% contribution to overall concentration). Manna quality (sucrose/raffinose) was strongly and positively associated with PC1 (r = 0.976, *p* < 0.001) but not with PC2 (r = 0.040, *p* = 0.884). Manna weight (mg) showed no significant correlation with either PC1 (r = 0.393, *p* = 0.132), PC2 (r = –0.001, *p* = 0.998), or manna quality (r = 0.3931, *p* = 0.1320).

### 2.2. Within-Tree Variation

**Figure 2:**
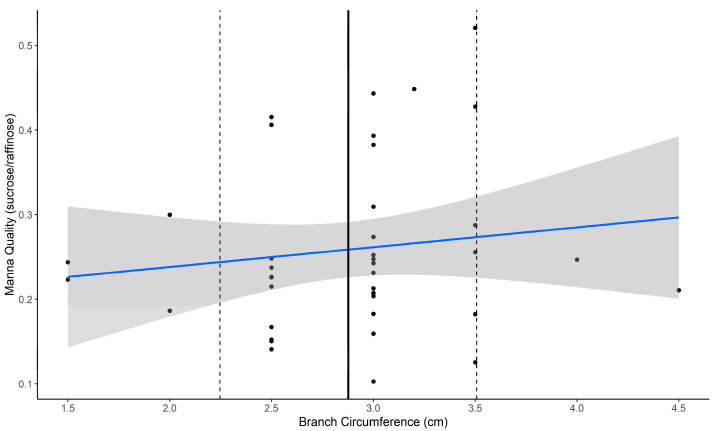
Relationship between branch circumference, (x-axis) and manna quality (y-axis) sampled during the November 2022 period, where the bold line represents the mean branch circumference (3 cm), the dashed line represents the standard deviation (±0.6), blue line is manna quality as estimated by linear regression (slope of 0.2880), and the shaded area depicts the 95% confidence interval.

Using data from 10 trees for which we had estimates of manna from multiple tree branches, we first explored the extent to which manna quality and quantity varied within trees in response to aspect, branch height, and branch circumference (Aim 1). Our results suggested that there was limited variation in manna *quantity* within a tree as a function of branch aspect, branch height, or branch circumference, (Table 2a). In contrast, manna *quality* varied with branch circumference (Table 2b). Specifically, branches with a larger circumference produced significantly higher quality manna, although the effect size for this relationship was relatively weak (Figure 2). There was no evidence that either branch aspect or branch height explained any further variation in manna quality (Table 2b). A likelihood ratio test comparing models with and without the random effect of tree ID indicated that its inclusion significantly improved model fit for both manna quality and quantity. This suggests that variation between individual trees contributes meaningfully to the observed variation in manna (Table 2).

### 2.3. Between-Tree Variation

Variation in manna quality and quantity between trees was assessed using data from the November (spring) 2022 sampling period, where we also collected data on tree health (vigor) and size (DBH) from our 30 *E. viminalis* trees (Aim 2). As multiple branches were collected per tree, we had a total of 60 observations from our thirty trees, and this replication was statistically controlled by including branches nested within trees as a random variable in our model (see Section 4).

**Figure 3:**
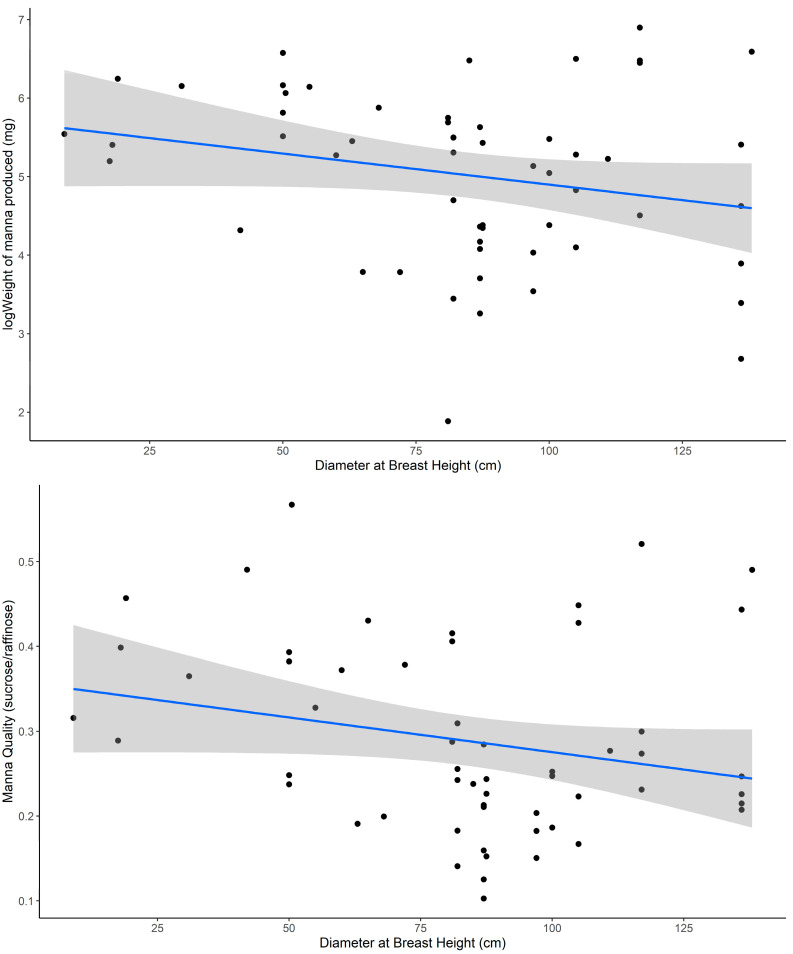
Relationship between manna quantity (**top**) and quality (**bottom**) with tree size (DBH). Solid lines represent linear regression estimates of manna quantity (**top**) and quality (**bottom**), with shaded areas indicating 95% confidence intervals (Coeff = −0.1910, CI = −0.6607, 0.2786 and Coeff = −0.1689, CI = −0.3902, 0.0525, respectively).

There was a significant negative relationship between both manna quantity and quality and tree DBH (Table 3). Specifically, larger trees (e.g., DBH > 50) produced lower quality manna and in smaller quantities (Figure 3). Tree health score (vigor) did not explain between-tree variation (Table 3).

### 2.4. Seasonal Variation

**Figure 4:**
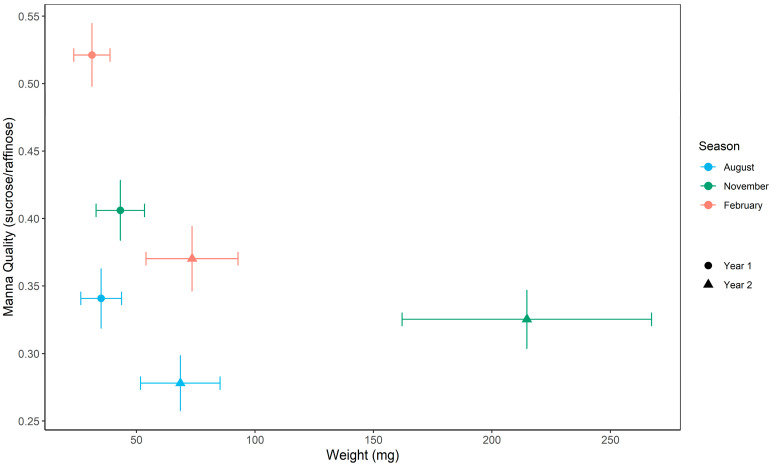
Least-squares means (LSMs) ± standard error (bars) for manna quality (sucrose/raffinose) and quantity (weight mg), derived from linear mixed models using the emmeans package [44]. LSMs represent adjusted means accounting for fixed effects of season and sampling year and a random effect of tree ID. Seasonally, August corresponds to winter, November to spring, and February to summer.

Manna quality and quantity varied significantly across sampling seasons and between years (Table 4, Figure 4). Quality was consistently highest in February, intermediate in November, and lowest in August, with significantly higher values in Year 1 than Year 2. In contrast, manna quantity peaked in November in both years and was substantially higher overall in Year 2. For example, estimated least-squares means (LSMs) for manna quality reached 0.521 ± 0.024 SE in February 2022 and dropped to 0.279 ± 0.021 SE in August 2022. Quantity peaked at 269.8 mg ± 63.56 SE in November 2022 compared to 43.2 mg ± 10.33 SE in November 2021. Despite this increase, the season × year interaction for manna quantity was not statistically significant (Table 4a).

Seasonal differences in temperature and rainfall may contribute to the variation observed above. To assess this, associations between manna traits and the monthly heat moisture index (a proxy for aridity) were also tested. Aridity was selected given the known influence of water availability and temperature on plant water relations and metabolic processes, including carbohydrate formation and accumulation (see Discussion). Manna quality showed a significant positive correlation with aridity (Coeff = 0.1654, *z* = 2.8830, and *p* value = 0.0039), indicating that higher sucrose/raffinose ratios were associated with drier conditions. In contrast, manna quantity showed no significant correlation with aridity (Coeff = −0.0637, *z* = −1.1098, and *p* value = 0.2671).

## 3. Discussion

We examined the within-tree and between-tree variation in manna quantity and quality of *E. viminalis*. Our results provide insight into trait-based variation in phloem exudate composition and how carbohydrate allocation strategies may shift across structural scales (branch vs. whole tree) and environmental conditions. Below, we discuss our results within the context of physiological mechanisms and adaptive strategies of *E. viminalis* that may be influencing variation in manna and consider future research directions and the broader implications of our results.

### 3.1. Manna Composition

Manna composition across the 30 *Eucalyptus viminalis* trees sampled was strongly dominated by sucrose and raffinose, which together made up approximately 95% of total sugar concentration. This pattern was consistent across sampling periods and aligns with previous findings on phloem exudates in eucalypts [19]. These two sugars also accounted for nearly all variation in the multivariate space (95% along PC1), indicating that shifts in their relative abundance are the primary source of functional variation in manna composition. Indeed, both sucrose and raffinose are well documented in their roles in carbon transport, storage, and physiological responses to stress [1,2,3,13,19,45,46].

In contrast, the remaining sugars (fructose, glucose, stachyose, and tetrasaccharides) contributed only a small proportion of total concentration (<5%) but showed subtle seasonal shifts in their multivariate positioning. These changes, detected visually and supported by the Procrustes analysis, likely reflect minor adjustments in internal carbon allocation or stress physiology [3,18,21,47]. Although the ecological roles of these minor sugars are less clear, their seasonal variation may reflect subtle shifts in stress physiology or biotic interactions [5,7,8,20] and could be more informative under broader environmental or spatial contexts.

### 3.2. Within-Tree Variation

We found that variation in manna quality, as measured by the sucrose-to-raffinose ratio, increased significantly with branch circumference, suggesting that localized branch traits such as size and leaf area influence sugar transport and allocation [31,32,34,48]. Thus, variation in manna quality within a single tree may be shaped by fine-scale differences in evaporative demand and carbohydrate assimilation capacity among branches [27,28,29]. Larger branches are likely to experience greater hydraulic and evaporative demand than smaller branches due to increased canopy leaf area [48]. Because leaves are the primary sites of carbohydrate assimilation, greater leaf area generally correlates with higher carbohydrate production [30,33]. Therefore, fine-scale variation in sink strength or hydraulic demand within an individual tree appears to influence branch level variation in manna quality [31,32,34,48]. Notably, manna quantity (weight mg) did not vary within trees.

### 3.3. Between-Tree Variation

Between trees, we observed a distinct pattern in how size influenced manna production. Tree size (DBH) is a key integrative trait that reflects both structural investment and physiological capacity and was a strong predictor of manna quantity and quality. Specifically, larger trees produced lower quality and quantity of manna. While carbohydrate assimilation generally increases with tree size due to expanded leaf area and greater metabolic activity [30,33], the strongest declines in manna output were observed in individuals above ~75 cm DBH. This negative association with DBH suggests a potential size threshold beyond which internal allocation strategies may shift, with larger trees potentially prioritizing carbon storage or maintenance over investment in phloem exudates. Trees with greater DBH are also more likely to have increased structural and hydraulic demands and may allocate more carbohydrates toward storage in woody tissues and roots as a protective strategy under environmental stress [2,35,36,37,38]. Allocation to belowground or perennial storage structures, including lignotubers, has been proposed as a resilience mechanism against both biotic (e.g., browsing) and abiotic (e.g., drought) stresses [49,50,51]. Eucalypts are known to demonstrate such resilience-type strategies by mobilizing stored sugars such as sucrose, glucose, and fructose to support recovery of hydraulic function following stress [49,51]. In this context, reduced manna production in large trees may reflect a shift in carbon allocation away from exudate outputs and toward the maintenance and resilience of essential tissues. Furthermore, increased hydraulic demand associated with maintaining larger biomass may constrain the capacity to allocate carbon toward non-essential outputs such as manna [52,53]. Ecologically, these findings suggest that mixed-age stands may offer the greatest benefit to dependent species by combining high-quality manna production from smaller to medium-sized trees with nesting opportunities provided by hollows in older, larger individuals [54]. This result highlights the broader ecological value of maintaining structural heterogeneity in *E. viminalis* stands, supporting both food availability and essential nesting habitat for associated fauna.

### 3.4. Seasonal Variation

There was strong seasonal and interannual variation in manna traits, with manna quantity highest in the spring and lowest in the winter and late summer. These patterns are consistent with seasonal differences in carbohydrate assimilation, where woody plants tend to maximize carbohydrate production during favorable growing periods and then allocate reserves to sink tissues for use during times of lower productivity or increased demand [1,2,4,12,36,37]. Such temporal variation likely influences not only the total carbon available for secondary outputs, such as exudates, but also the specific sugars mobilized across seasons. The seasonal patterns observed are potentially linked to climatic factors, particularly water availability and evaporative demand, which affect the physiological mechanisms described above. In support of this, we detected a significant association between manna quality (as measured by the sucrose/raffinose ratio) and aridity, represented by the monthly heat–moisture index. This association between manna quality and aridity aligns with prior evidence that sucrose and raffinose concentrations in phloem sap respond differentially to water availability [4,11,12,45]. In *Eucalyptus globulus*, for example, wetter conditions have been linked to lower sucrose concentrations, potentially reducing manna quality, while drier conditions may elevate sucrose expression and improve quality [4,11,12,45]. As osmoregulatory sugars, sucrose and raffinose help maintain cell function under water stress and temperature extremes [3,18,45,46,47,55].

The seasonal shifts we observed in exudate composition may therefore reflect flexible carbohydrate partitioning strategies in response to rainfall and water availability [38,49,52], with potential consequences for both plant resilience and resource partitioning [50,51]. However, this climate association was drawn from a single correlation within a limited dataset and should be interpreted cautiously. Nonetheless, it points to an intriguing direction for future research on climate influences on manna traits.

## 4. Materials and Methods

### 4.1. Manna Collection and Extraction

This study was conducted at the Queens Domain reserve, in southeast Tasmania (−42.87, 147.32), 80 m above sea level. This is a relatively dry, temperate site, experiencing mean annual rainfall of 571 mm, lowest temperature of 7.8 °C, and highest temperature of 17 °C (Bureau of Meteorology “Climate Data” portal 2024). Thirty adult *E. viminalis* trees ranging in size (diameter at breast height [DBH] 9–138 cm) were repeatedly sampled from for manna over three seasons (August = winter, November = spring, and February = summer) across three years (2021–2023). Although samples were collected across three calendar years, data were grouped into two sampling years based on seasonal sampling periods: Sampling Year 1 includes August 2021, November 2021, and February 2022; Sampling Year 2 includes August 2022, November 2022, and February 2023. This reserve land contains patches of both modified (mowed and cleared) and native vegetation. The stand in which our 30 trees were selected is classified as *E. viminalis* woodland, with a grassy understory containing a mix of native and exotic species. Here, *E. viminalis* is the dominant tree species within a medium-density mixed-age stand comprised of juvenile to established adult trees (up to 10 m tall, DBH 138 cm).

**Figure 5:**
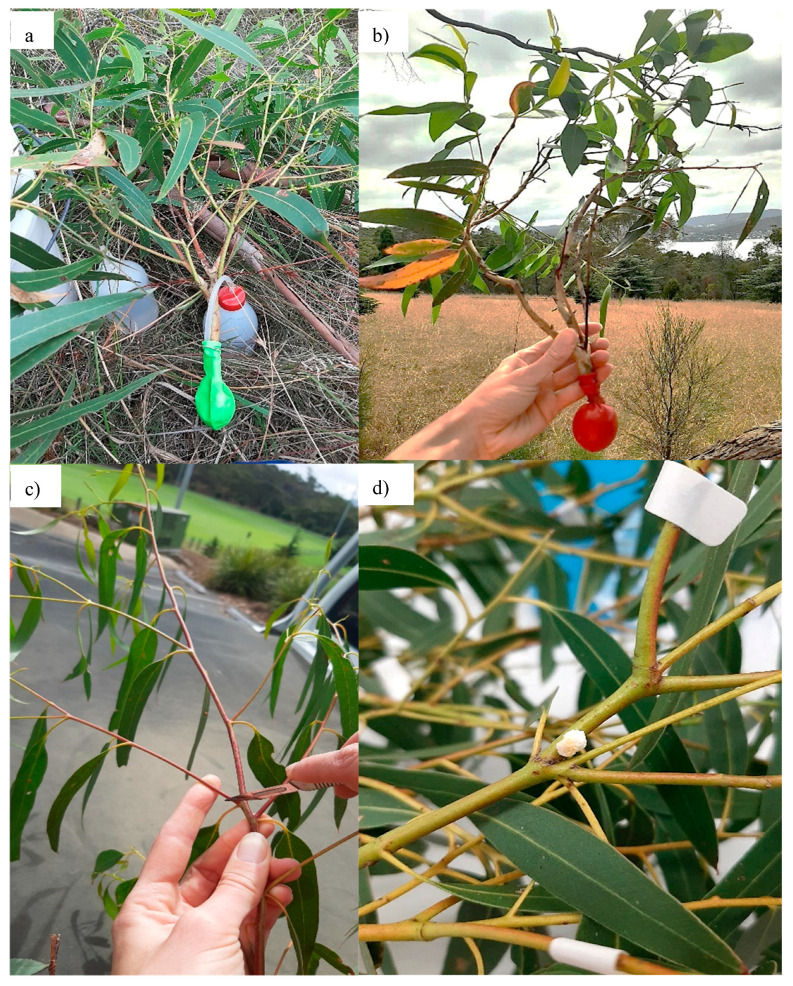
Images of branch collection and branch incision process. (**a**) Attaching balloon to prevent water loss, (**b**) typical “branch” with balloon attached, (**c**) example of incision site at stem-branch junction, and (**d**) image of crystalized manna 24 h after incision.

Full details on the sampling methodologies are outlined in Appendix A.1. Briefly, a single branch ranging between 1.5 and 4.5 cm in circumference and 30 and 50 cm long was collected per *E. viminalis* tree using pruning poles from between 1 and 7 m into the canopy. Upon collection, branches were placed in a balloon that was then filled with water using a safety wash bottle and sealed with a rubber band to prevent water loss (see Figure 5). DBH was measured for each tree as a proxy for tree size.

Sampling protocols were as above for all sampling periods except for the November (spring) 2022 period. During this period, we selected 10 of our 30 trees to investigate within-tree variation in manna quantity and quality (Aim 1). Here we collected up to five branches from each tree to test whether within-tree variation is influenced by factors related to branch selection, which could be standardized in the sampling protocol. These factors included i) the approximate height of the branch within the canopy (<4 m for low, >5 m for high), ii) branch circumference (measured to the nearest 0.5 cm), and iii) aspect of the tree from which the branch was collected (N, S, E, or W)

We also collected data relating to tree health to explore the effect of tree health on manna quality and quantity (Aim 2). Here, we created an overall health score (vigor) based on established canopy damage assessment traits [56,57]. Specifically, vigor was calculated as the percentage of the whole tree canopy that was dead (evidence of dieback), plus the percentage of foliar damage by insects and pathogens for the branch collected. Here, a lower vigor score was considered indicative of a “healthier” tree, and a higher score as indicative of more canopy damage.

Branches were housed on campus at the University of Tasmania and kept at room temperature in shaded conditions. On arrival, balloons were removed, and branches immediately placed into pre-prepared jars of water (0.5–3 L volume). Approximately 2 cm of the stem was cut from the base of each branch to remove dysfunctional xylem cells that may have filled with air during the collection process, enabling plants to maintain hydration during manna extraction. Using a scalpel, 8–12 incisions per stem were made evenly in the leaf/stem axils, (as dictated by the number of leaves/stem axils per stem) across at least six stems of each branch (or all stems where there were less than six per branch).

After 24 h, incisions were checked for manna. If manna appeared transparent, collection was delayed, allowing time for crystallization up to a maximum of 76 h, (Figure 5d). If manna was not present 24 h after collection, an additional 6–10 incisions were made per branch, and sample collection was delayed for another 24 h (48 h in total). Once crystalized, manna was collected from each branch by gently scraping crystallized secretions, using a scalpel, into a 10 mL vial labeled with the corresponding branch identification number (see Appendixes A.1 and B.1 for method development details). The quantity of manna produced per branch was determined using a Mettler Toledo (XPE105DR) precision balance (Mettler-Toledo, Greifensee, Switzerland) as total amount (weight in milligrams to four decimal places) produced over a 48 h period.

### 4.2. Manna Chemical Analysis

Once weighed, manna quality was quantified using Liquid Chromatography–Mass Spectrometry (LC-MS). To prepare samples for LC-MS analysis a subsample of manna (0.01 mg min–2.00 mg max) was placed into a 2 mL Autosampler vial (Waters, Milford, MA, USA). Manna was then dissolved with equal proportions of distilled water (e.g., 1 mg of manna with 1 mL of distilled water), and the vial was vortexed for 20 s until manna was dissolved. All vials were stored in −20 °C conditions to limit deterioration until analysis.

LC-MS was conducted at the University of Tasmania’s Central Science Laboratory using a Waters Acquity H-Class UPLC instrument coupled to a Waters Xevo TQ triple quadrupole mass spectrometer to determine manna composition and concentration of sugars present in each sample as per Wing [41]. Briefly, the mass spectrometer was operated under negative ion electrospray mode with a needle voltage of 2.7 kV. Analytes were detected using single-ion monitoring with [M-H]-deprotonated molecular species. Data were processed using MassLynx software version 4.2 (Waters, USA). Individual sugars (raffinose, stachyose, glucose, fructose, and sucrose) were identified and quantified (into parts per million, PPM) using the external calibration approach. Unidentified tetrasaccharides were grouped and quantified as “Tetrasaccharides” (see Appendix C.1).

### 4.3. Data Analysis

All analyses were performed using R version 4.4.0 [58], and figures were generated using base R and ggplot2 [59].

#### 4.3.1. Characterizing Manna Composition and Quality

Manna composition is defined here as the profile of the six major sugars present in *Eucalyptus viminalis* manna (sucrose, raffinose, fructose, glucose, stachyose, and tetrasaccharides), characterized by their absolute concentrations (PPM), proportional contributions to total sugar content, and patterns of variation in the multivariate space (PCA).

To analyze manna composition, we first conducted bivariate outlier tests to visualize the distributions of the six manna sugars and identify potential outliers. We then calculated the Mahalanobis distance for each observation and assessed whether each distance value was statistically extreme by calculating *p* values for each point based on a chi-squared distribution, using the “qchisq” function [58]. A significance level of 0.001 was chosen to identify extreme outliers while retaining moderate variability in the dataset for subsequent analyses. Identified outliers were removed from further analysis (see Appendix D.1). The mean absolute concentration (PPM) and proportional contributions to the total sugar content for the six manna sugars are reported (Table 1).

We used principal component analysis (PCA) using the “prcomp” function [58] to reduce the complexity of the six correlated manna sugars into two informative axes that capture the main patterns of variation in manna composition within the 30 *E. viminalis* trees sampled. This multivariate approach allowed us to summarize how the six sugars co-vary and identify dominant sugar groupings. We used PCA exclusively on the six sugars to explore variation in manna composition, not to identify environmental or tree-level drivers, which were tested separately.

To prepare sugar data for the PCA, data were first transformed to reflect the proportion contribution to the overall sample concentration to account for volumetric variance, (e.g., influence of sample volume on sugar concentration and composition). As proportional data do not meet the assumptions of linearity required for principal component analysis, we applied an arc-sine transformation [60]. We first ran PCA on the November 2022 manna data, as this corresponded to the sampling period that included our data for within-tree variation and health (see Results, Figure 1).

To explore how correlations between the six key sugars—raffinose, sucrose, fructose, glucose, stachyose, and tetrasaccharides—and the principal component axes varied across sampling periods, we used the “vegan” package [61] to vector fit each sampling event, grouped by season and year, into the PCA space defined by the November/spring 2022 data (PC axes 1 and 2). We then performed Procrustes pairwise tests within seasons (e.g., August 2021/August 2022) and in comparison to the reference period (November 2022) to consider the degree of variation in the spatial configuration of the sugars predicted into the multivariate space (see Appendix E.1).

Across the experimental period, each manna sample was mostly comprised of two key sugars, sucrose and raffinose (see Results). These sugars have been proposed to reflect differing roles in plant carbohydrate storage and allocation and may also influence how exudates are utilized by foraging fauna [7,8,20,21,26,47]. To confirm that the ratio of these two dominant sugars captures the variation in manna quality generated from our PCA, we conducted a Pearson’s correlation test between our first two principal components and the ratio of sucrose to raffinose using the “corr.test” function [58]. Given that sucrose and raffinose are equally represented by PC1, show a significant correlation with this axis, and are known to make up ~90% of manna composition [19], we hereafter used the ratio of sucrose/raffinose as our indicator of manna quality. Here, a higher ratio value indicates a greater proportion of sucrose in a sample, indicative of increased quality.

#### 4.3.2. Variation in Manna Quality and Quantity

Histograms were generated to visualize the distribution of manna quality (sucrose ratio) and quantity (weight in mg). Outliers were removed where the sucrose/raffinose exceeded 1 and manna weight exceeded 1000 mg. While these data points were genuine observations, their values fell beyond three standard deviations for quality (0.3316, ± 0.1348) and quantity (143.46 mg ± 205.49 mg) in the full dataset, rendering them unrepresentative of the overall trends (see Appendix D.1).

Models exploring the within- and between-tree variation in manna utilized the November/spring 2022 datasets relating to branch sampling traits (aspect, height, and branch circumference) and tree health (vigor). First, using a subset of trees for which we had data from multiple branches across the tree, we explored whether manna quality and quantity varied within a tree as a function of key branch traits. To achieve this, we ran a linear mixed-effects model using the “glmmtmb” package [62], with manna quality or quantity as the response variable and branch aspect, branch height, and branch circumference as predictor variables, where tree ID was retained as a random variable. Manna quantity data (weight) was log transformed to normalize data and family set to “gaussian”. As the data for manna quality (sucrose/raffinose) were proportional and beta distributed, the “beta” family with the “logit” link was applied. Restricted maximum likelihood (REML) was set to true for both models to account for the unbalanced replication in branch number. The “DHARMa” package was used to test model assumptions and fit (manna quality and quantity) using the simulateResidual and testDisperson functions [63]. Type III Wald chi-square tests were calculated for model fixed effects using car: Anova [64]. Variation attributed to random effects was approximated using Wald likelihood ratio tests (LRTs), comparing models with and without the random effect. This modeling approach was applied for all other models detailed below.

Second, we explored whether manna quality and quantity varied between trees as a function of tree health. To achieve this, we ran a linear mixed-effects model with manna quality or quantity as the response variable and tree vigor score and tree size (measured as DBH) as predictor variables. Here, branch ID was nested within-tree ID as a random effect to account for the unequal replication of branches sampled per tree.

#### 4.3.3. Seasonal Variation in Manna Quality and Quantity

To investigate the extent of seasonal and annual variation in manna we ran linear mixed-effects models with manna quality (sucrose/raffinose) and quantity (weight mg) as response variables, sampling season (August, November, and February), year, and their interaction as fixed effects, and tree ID as a random variable. To account for the fact that we sampled multiple branches per tree within some sampling periods, we generated estimates of the mean manna quality and quantity per tree per sampling period.

The model for manna quality showed a non-significant within-group deviation with Levene’s test for homogeneity. Further investigations identified a single individual (tree 18) as the cause of this deviation. We then ran the model with and without tree 18 and found no difference in the significance or interpretation of the relationship; thus, the dataset inclusive of tree 18 was utilized.

To explore whether the seasonal variation we observed was related to climatic variation, we calculated a proxy for aridity based on the annual heat moisture index (AHM). The AHM has previously been used to examine climate-based variation in *Eucalyptus* and is known to show associations with plant traits such as height and DBH [43,65,66].

Here, we calculated a monthly heat moisture index per sampling period using the mean rainfall (mm) and mean highest temperature (°C) [TempH] for the month of sampling, extracted from the Bureau of Meteorology “Climate Data” portal (Commonwealth of Australia 2022). The monthly heat moisture index was calculated as:

Monthly heat moisture index = (mean TempH + 10)/(mean Rainfall/1000)(1)

To explore seasonal variation in manna, we correlated the monthly heat moisture index against manna quality and quantity by performing a Kendall’s rank correlation using the corr.test function. Given the sample size (six sampling events across two years), the results should be interpreted with caution.

Least-squares means (LSMs) and standard errors were extracted from the final model using the emmeans package in R [44] and used to visualize seasonal and annual trends in manna quality and quantity, (see Results, Figure 4).

## 5. Conclusions

Our results highlight both within- and between-tree variation in manna quantity and quality in *Eucalyptus viminalis*, with two key sugars, sucrose and raffinose, driving the major axes of compositional variability. The observed temporal and within-population variation is consistent with our hypothesis that differences in carbohydrate allocation, storage, and assimilation underlie variation in manna expression [1,2,4,12,36,37], driven by fine-scale variation associated with branch circumference and tree size (DBH), combined with temporal and climatic influences. These dynamics may have downstream consequences for resource availability within forest ecosystems, particularly for species that rely on manna as a food source. Although tree health did not significantly predict manna variation, the impact of canopy decline or dieback is an important area for future research, especially for considering impacts on manna production in the context of broader ecosystem resource flows [67]. Understanding the effects of temporal and within-population variation on manna is important not only for anticipating ecological responses to environmental change but also for advancing our knowledge of how foundational plant species regulate carbohydrate partitioning under varying physiological and climatic conditions.

## Figures and Tables

**Table 1:** Mean value for manna sugar composition of each of the six main sugars found in manna in parts per million (mean) and percentage contribution of each sugar to the total concentration (% total) for 248 branches (n) sampled from the 30 *E. viminalis* trees across the experimental period (2021–2023).

	Mean (ppm)	±SE	% Total
Raffinose	603.33	9.43	71.73
Sucrose	214.66	5.26	25.15
Stachyose	18.64	0.6	2.2
Fructose	8.72	0.55	0.57
Glucose	1.77	0.25	0.21
Tetrasaccharides	1.17	0.18	0.14

**Table 2:** Results from Type III Wald chi-square tests and LRT for associated random effects from linear mixed models investigating variation in manna (**a**) quantity (weight mg) and (**b**) quality (sucrose/raffinose) with branch collection traits (aspect, height, and branch circumference).

**(a) Quantity**	**chisq**	**df**	***p* Value**
Aspect	4.0064	3	0.2608
Height	1.5372	2	0.4637
Branch circ	0.0645	1	0.7995
Random effects	*LRT*	*df*	*p* value
*(1|tree ID)*	3.18	1	0.037
**(b) Quality**	**chisq**	**df**	***p* Value**
Aspect	2.7229	3	0.4364
Height	0.0831	2	0.9593
Branch circ	3.8664	1	0.0493
Random effects	*LRT*	*df*	*p* value
*(1|tree ID)*	6.28	1	0.006

**Table 3:** Results from Type III Wald chi-square tests and LRT for associated random effects from linear mixed models investigating between-tree variation in manna (**a**) quantity (weight mg) and (**b**) quality (sucrose/raffinose) with tree health (vigor) and size (DBH). Given the relationship identified above, branch circumference (branch circ) was also included as a covariate.

**(a) Quantity**	**chisq**	**df**	***p* Value**
Vigor	2.7892	1	0.0949
DBH	4.5465	1	0.0330
Branch circ	0.6355	1	0.4254
Random effects	*LRT*	*df*	*p* value
*(1|branch ID: tree ID)*	0	1	0.5
**(b) Quality**	**chisq**	**df**	***p* Value**
Vigor	3.1147	1	0.0776
DBH	4.9988	1	0.0254
Branch circ	2.2364	1	0.1349
Random effects	*LRT*	*df*	*p* value
*(1|branch ID: tree ID)*	0	1	0.5

**Table 4:** Results from Type III Wald chi-square tests and LRT for associated random effects from linear mixed models investigating between variation in manna (**a**) quantity (weight mg) and (**b**) quality (sucrose/raffinose) across sampling season (August, November, and February) and years (Sampling Year 1 = 2021–2022 and Sampling Year 2 = 2022–2023).

**(a) Quantity**	**chisq**	**df**	***p* Value**
Season	12.108	2	0.002
Year	30.601	1	<0.001
Season × Year	4.7341	2	0.094
Random effects	*LRT*	*df*	*p* value
*(1|tree ID)*	4.86	1	0.014
**(b) Quality**	**chisq**	**df**	***p* Value**
Season	41.474	2	<0.001
Year	33.0653	1	<0.001
Season × Year	3.6325	2	0.163
Random effects	*LRT*	*df*	*p* value
*(1|tree ID)*	8.06	1	0.002

**Figure A1:**
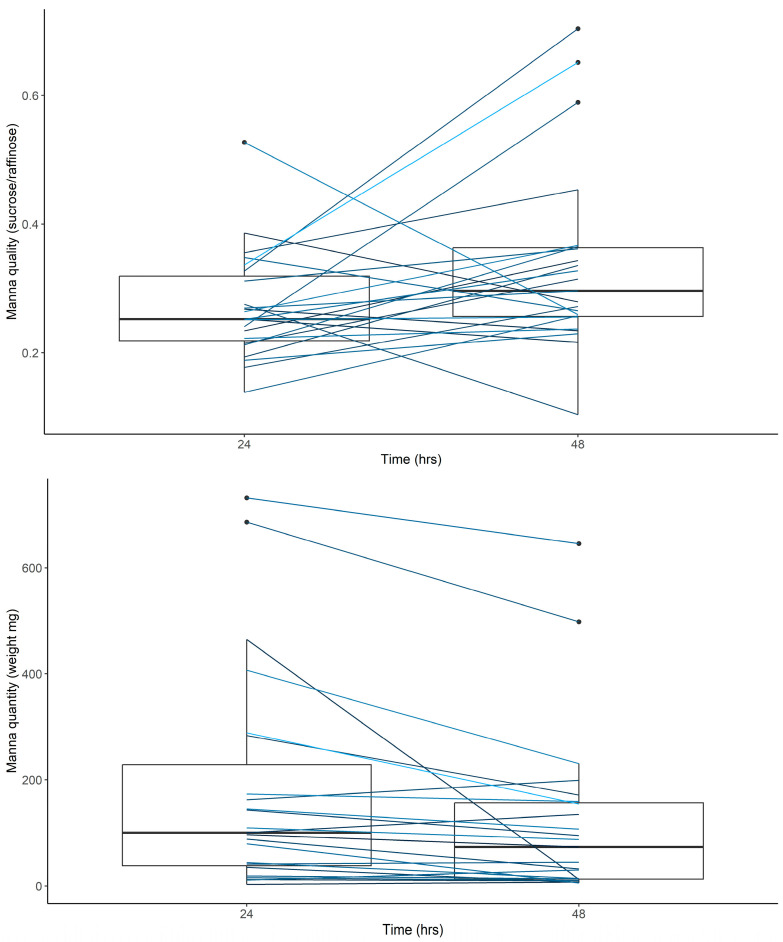
Boxplot showing variance in manna quantity (**top**) and quality (**bottom**) between the 24 and 48 h collection periods. Points represent individual trees, connected by a blue line to illustrate within-tree variation.

## Data Availability

Data will be made available upon acceptance of publication via the University of Tasmania Research Data Portal.

## Abbreviations

The following abbreviations are used in this manuscript:

LC-MS	Liquid Chromatography–Mass Spectrometry
DBH	Diameter at Breast Height
AHM	Annual Heat Moisture Index
PCA/PC	Principal Component Analysis/Principal Component
LRT	Likelihood Ratio Test
LSMs	Least-Squares Means

## Appendix A

### Appendix A.1. Manna Sampling Procedure Method Development

To devise a successful method of ex situ manna extraction, we conducted several preliminary trials to determine the optimal number of incisions, location of incisions, branch circumference, extraction time, and hydration medium. We also considered if time between collection and first incisions influenced manna production (travel time) (see below). Manna production was considered as either a success when manna crystals were present or failure if no crystals were detected.

#### Preliminary Branch Selection and Collection Trials

We tested the difference in manna production between younger (newly expanded) and mature stem growth. Younger stems were characterized as olive green to red in appearance and positioned toward the branch tips, versus the mature woody stems.

The circumference and length of each branch was recorded to the nearest 0.5 cm using a measuring tape, and a minimum branch length of 30 cm was determined.

We considered three branch transport mediums: balloons (27.5 cm latex helium quality), floral foam brick, and 20 L bucket filled with water. Upon collection, branches were either placed in a balloon filled with water using a 250 mL safety wash bottle and sealed with a medium-sized rubber band to prevent water loss, placed in a hydrated 23 × 11 × 8 cm brick of floral foam at 6–8 branches per brick, or placed directly into a 20 L bucket filled up to 5 L with water.

A trial was considered successful if manna was present at 24, 48, or 76 h. The degree of success was visually determined by the presence of absence of manna nodules/secretions (see Table A1 for detailed trial successes).

**Table A1:** Matrix of trial factors considered for manna collection and extraction. Here, x represents success, and N/A indicates factor overlap. Factors that yielded zero successes, e.g., mature stems, are not included.

	No. Incisions		Branch Type	Hydration Medium			Extraction Time (h)			Branch Circumference		Travel Time (h)	Travel Time (h) with Initial Stimulation		
**Factors trialed**	12	20	Young growth	Balloon	Floral foam	Bucket	24	48	76	1–2 cm	>3 cm	4	8	6	4
12	N/A	N/A	x	x		x	x	x	x	x	x	x	x	x	x
20	N/A	N/A	x	x	x	x	x	x	x	x	x	x	x	x	x
New growth	x	x	N/A	x	x	x	x	x	x	x	x	x	x	x	x
Balloon	x	x	x	N/A	N/A	N/A	x	x	x	x	x				
Florists’ foam		x	x	N/A	N/A	N/A									
Bucket	x	x	x	N/A	N/A	N/A									
24	x	x	x	x	x	x	N/A	N/A	N/A		x	x	x	x	x
48	x	x	x	x	x	x	N/A	N/A	N/A	x	x	x	x	x	x
76	x	x	x	x	x	x	N/A	N/A	N/A		x				
1–2 cm	x	x	x	x		x	x	x	x	N/A	N/A				
>3 cm	x	x	x	x		x	x	x	x	N/A	N/A	x	x	x	x
8		x	x	x			x	x	x		x	N/A	N/A	N/A	N/A
6	x	x	x	x			x	x	x		x	N/A	N/A	N/A	N/A
4	x	x	x	x			x	x	x		x	N/A	N/A	N/A	N/A

Note: if travel time from field to laboratory is under 4 h and branch hydration is maintained, field incisions are not necessary.

If manna was not present 24 h after collection, an additional 6–10 incisions were made per branch, and sample collection was delayed for another 24 h (48 h in total). If manna appeared transparent, collection was delayed, allowing time for crystallization for a maximum of 76 h.

The results from these trials suggested that branch hydration and travel time greatly contribute to the success of manna extraction and, thus, the quantity of manna produced. Therefore, if initial incisions are made at the time of collection or within four hours of collection and individual branch hydration is maintained throughout the extraction and collection process, the quantity of manna produced is unlikely to be influenced.

## Appendix B

### Appendix B.1. Variance in Manna Composition with Collection Period

Data from the winter 2022 collection period was used to determine variance in manna quality and quantity between the two collection periods (24 h vs. 48 h). Here, manna was collected from branches that successfully produced manna at 24 h and again at 48 h. While some observable variation is present between manna quality and quantity and the two collection times (Figure A1), an analysis of variance demonstrated that this variation was not of statistical significance for both manna quality and quantity across the two collection periods (*ANOVA*, F_1,44_= 3.453, *p* = 0.0699 and F_1,44_= 1.229, *p* = 0.2737, respectively), suggesting that manna collected at either period adequately represents manna productivity within the individual plant sampled.

## Appendix C

### Appendix C.1. LC-MS Calibration

LC-MS was conducted at the University of Tasmania’s Central Science Laboratory, using a Waters Acquity H-Class UPLC instrument coupled to a Waters Xevo TQ triple quadrupole mass spectrometer. A Waters Acquity UPLC BEH Amide column (50 × 2.1 mm, 1.7 um) was used, and the mobile phase used two solvents of 0.4% (*v*/*v*) Ammonium hydroxide in water (solvent A) and Acetonitrile (solvent B). The UPLC program ran for 6 min at 20% A:80% B to 45% A:55% B, which was followed by immediate re-equilibration to starting conditions for three minutes. The column was held at 50 °C and the sample compartment at 6 °C. The flow rate was 0.35 mL min^−1^ with an injection volume of 1 uL.

The mass spectrometer was operated under negative ion electrospray mode with a needle voltage of 2.7 kV. Analytes were detected using single-ion monitoring with [M-H]- deprotonated molecular species. To detect C6 monosaccharides, (m/z) 179.05 with a cone voltage of 17 V was used. For disaccharides, (m/z) 341.10 at 24 V; trisaccharides, (m/z) 503.15 at 30 V; and, finally, tetrasaccharides, (m/z) 665.20 at 36 V were used. The dwell time was 0.057 s and the ion source temperature 130 °C. Desolvation gas was N2 at 950 L h^−1^ (with a temperature of 450 °C), and the cone gas flow was 100 L h^−1^. Data were processed using MassLynx software (Nichols, D. pers comm). Data from the winter 2022 collection period was used to determine variance in manna quality and quantity between the two.

## Appendix D

### Appendix D.1. Determination of Outliers

**Table A2:** Outliers for manna sugar composition (fructose, glucose, sucrose, raffinose, stachyose, and tetrasaccharides) identified by Mahalanobois distance and chi-squared distribution removed from dataset. Sugars are in parts per million (PPM).

Year	Season	Tree ID	Fructose	Glucose	Sucrose	Raffinose	Stachyose	Tetra
2021–2022	Spring	18	23.77	17.08	182.82	680.07	33.57	0.00
2021–2022	Spring	19	0.00	0.00	588.26	1113.31	50.66	0.00
2021–2022	Spring	20	10.45	11.75	273.67	691.22	44.77	0.00
2021–2022	Spring	22	16.47	12.31	50.27	743.31	4.30	3.28
2021–2022	Spring	28	47.89	39.47	90.03	591.03	14.22	0.00
2021–2022	Summer	2	6.94	2.55	159.88	406.23	18.99	26.10
2021–2022	Summer	21	36.84	30.94	136.43	476.81	31.13	26.66
2021–2022	Summer	25	4.56	3.38	49.60	131.79	6.48	0.54
2022–2023	Summer	7	68.46	3.84	294.26	544.55	15.06	3.33
2022–2023	Summer	10	72.10	4.40	254.63	786.40	9.57	6.74
2022–2023	Summer	11	42.51	2.55	301.34	790.81	20.71	5.07
2022–2023	Summer	13	6.10	13.76	171.87	556.83	9.95	0.29
2022–2023	Summer	24	51.85	9.59	433.89	823.75	12.03	18.66
2022–2023	Summer	28	16.80	3.03	603.76	0.17	19.98	6.60
2021–2022	Winter	21	10.19	3.69	101.69	471.45	8.27	12.55
2021–2022	Winter	25	1.82	2.77	319.60	727.41	55.29	1.10
2022–2023	Winter	5	9.06	0.00	172.25	682.36	15.86	0.50

**Table A3:** Outliers identified for manna quality (ratio of sucrose:/raffinose) and quantity (weight in milligrams), where sucrose/raffinose was greater than one and the weight of manna produced exceeded 1000 mg.

Year	Season	Tree ID	Quantity(mg)	Quality
2021–2022	Winter	11	2.7	1.0133
2022–2023	Spring	29	2688.8	0.4437
2022–2023	Spring	1	1279.1	0.7281
2022–2023	Spring	6	1219.9	0.4403
2022–2023	Spring	30	1072.2	0.2843

## Appendix E

### Appendix E.1. Procrustes Pairwise Test Output

**Table A4:** Results of the Procrustes pairwise test comparing sugar configurations across all sampling periods predicted into the November 2022 multivariate space. The Procrustes sum of squares (m^2^) quantifies the dissimilarity between the configurations relative to the reference period (November 2022), while the correlation value indicates the degree of alignment between the configurations.

	m^2^	Correlation	*p* Value
August 2021	0.964	0.191	0.678
August 2022	0.747	0.503	0.036
November 2021	0.929	0.266	0.396
February 2022	0.788	0.461	0.067
February 2023	0.798	0.450	0.070
